# Macrospin dynamics in antiferromagnets triggered by sub-20 femtosecond injection of nanomagnons

**DOI:** 10.1038/ncomms10645

**Published:** 2016-02-05

**Authors:** D. Bossini, S. Dal Conte, Y. Hashimoto, A. Secchi, R. V. Pisarev, Th. Rasing, G. Cerullo, A. V. Kimel

**Affiliations:** 1 Institute for Molecules and Materials, Spectroscopy of Solids and Interfaces, Radboud University, Heyendaalseweg 135, 6525 AJ Nijmegen, The Netherlands; 2 Dipartimento di Fisica, Politecnico di Milano, Piazza Leonardo da Vinci 32, I-20133 Milano, Italy; 3 Istituto di Fotonica e Nanotecnologie, Consiglio Nazionale delle Ricerche, Piazza Leonardo da Vinci 32, I-20133 Milano, Italy; 4 Ioffe Physical-Technical Institute, Ferroics Physics Laboratory, Russian Academy of Sciences, St Petersburg 194021, Russia

## Abstract

The understanding of how the sub-nanoscale exchange interaction evolves in macroscale correlations and ordered phases of matter, such as magnetism and superconductivity, requires to bridging the quantum and classical worlds. This monumental challenge has so far only been achieved for systems close to their thermodynamical equilibrium. Here we follow in real time the ultrafast dynamics of the macroscale magnetic order parameter in the Heisenberg antiferromagnet KNiF_3_ triggered by the impulsive optical generation of spin excitations with the shortest possible nanometre wavelength and femtosecond period. Our magneto-optical pump–probe experiments also demonstrate the coherent manipulation of the phase and amplitude of these femtosecond nanomagnons, whose frequencies are defined by the exchange energy. These findings open up opportunities for fundamental research on the role of short-wavelength spin excitations in magnetism and strongly correlated materials; they also suggest that nanospintronics and nanomagnonics can employ coherently controllable spin waves with frequencies in the 20 THz domain.

## Abstract (web summary)

Magnetic order in nature is defined by the exchange interaction, however its understanding on the ultrafast- and nanometer-scales is limited. Here, the authors follow the femtosecond sub-nanometer dynamics of the antiferromagnetic order parameter in KNiF_3_ triggered by photo-generated exchange magnons.

## Graphical abstract

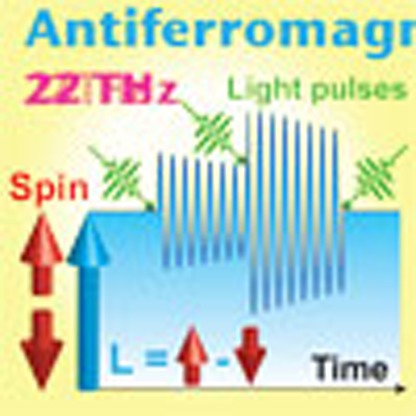

Experimental studies allowing to investigate correlated matter in general, and magnetism in particular, at the sub-nanometre length- and femtosecond timescales of the exchange interaction have recently developed into an exciting research area. The experiments involving ultrashort timescales provided intriguing results, like the femtosecond laser-induced transient ferromagnetic state of a ferrimagnet alloy^1^ and even light-induced superconductivity^2^. However, in these cases the wavelengths of the photo-induced excitations lie orders of magnitude above the lengthscale of the exchange interaction. An alternative strategy consists in the investigation of the magnetic order induced by introducing impurities with atomic resolution in space^3^,^4^,^5^, but without temporal resolution. A hitherto unexplored approach to the problem, which combines the femtosecond timescale with the nanometre lengthscale, consists in studying the ultrafast dynamics of the macroscale magnetic order parameter triggered by spin excitations with wavelength and period matching the length- and timescales of the exchange interaction.

These spin excitations correspond to magnons (or spin waves) with wavevector near the edges of the first Brillouin zone. In antiferromagnetic materials these magnons can be elegantly excited in the time domain, via a second-order impulsive stimulated Raman scattering process involving pairs of magnons with wavevectors almost equal in magnitude and opposite in sign. It was demonstrated that in the absence of an external magnetic field, such spin excitations can be generated only by a modification of the exchange interaction^6^ and cannot be triggered by a change of the spin–orbit coupling. A light-induced modification of the exchange interaction generates pairs of magnons (as shown in the Methods section) and properly describes the Raman spectra^6^,^7^. The microscopic mechanism of perturbation of the exchange interaction by a laser pulse has been recently described in terms of an effective energy shift of the charge-transfer transition^8^,^9^. Although pairs of spin waves could in principle be generated throughout the whole Brillouin zone, the magnon density of states is largest in the high-frequency region near the zone edges, where the frequencies are defined mostly by the exchange interaction^6^,^7^ (Supplementary Note 1 and Supplementary Fig. 1). Note that the wavelength of these magnons is comparable to the distance of nearest-neighbour spins, whose interactions determine the macroscopic magnetic order. Therefore, studying the photo-induced dynamics of such magnons discloses the response of the magnetic system to excitations on the length- and timescales of the exchange interaction. A bound state of two high-energy, high-wavevector and counter-propagating spin waves, usually denoted as two-magnon (2M) mode, can be induced by a femtosecond light pulse. The frequency and wavevector of this magnetic excitation are the sums of the frequencies and wavevectors of the two magnons involved in the bound state^6^,^7^,^10^,^11^. Although an impulsive excitation of the 2M mode was reported, the subsequent dynamics of the magnetic order parameter has not been discussed^12^. A previous attempt to measure the light-induced evolution of the 2M mode directly in the frequency domain, via the femtosecond-stimulated Raman scattering technique, could reveal a modification of the Raman shift of the 2M peak only during the overlap of the laser pulses^13^. This signal is dominated by nonlinear purely optical effects, originating from the simultaneous interaction of three ultrashort intense laser pulses with the sample. Therefore, disentangling the optical and the magnetic components of the signal was possible only via a sophisticated data analysis and modelling. In femtosecond-stimulated Raman scattering no signal at delays subsequent the instrument response time was observed, which did not allow to disclose the dynamical response of the magnetic order to the 2M excitation.

The present work reports the femtosecond dynamics of the macroscopic magnetic order parameter, by means of an impulsive all-optical injection of the intrinsically highest-frequency spin excitations with wavevectors near the edges of the Brillouin zone. The wavelengths of the corresponding magnons are of the order of 1 nm, and the frequencies in the 20 THz range. We demonstrate the manipulation of the order parameter via the coherent control of the short-range magnons: by changing the conditions of the photo-excitation we arbitrarily reverse the phase of the spin waves and enhance or quench their amplitude. Our time-resolved approach discloses even a dynamical coupling of the high-wavevector magnons with a lattice vibrational mode.

## Results

### Experimental approach

An excellent system for the all-optical excitation and detection of the dynamics of high-frequency and shortest-wavelength magnons is the cubic Heisenberg antiferromagnet KNiF_3_, which is ordered below the Néel point *T*_N_=246 K. In this material the Raman cross-section of the 2M mode is so high that it dominates the whole spectrum^6^,^14^. A time-domain excitation of the 2M mode in KNiF_3_ (*ν*_2M_≈22 THz, period≈45 fs, wavevector≈10^7^ cm^−1^ and wavelength≈1 nm) can be achieved by the impulsive stimulated Raman scattering mechanism, provided that the duration of the stimulus is shorter than the period of the magnetic mode^15^,^16^. A successful impulsive excitation of such high-frequency magnons therefore demands laser pulses with a duration significantly shorter than 40 fs. To meet these requirements we used linearly polarized sub-20-fs laser pulses, with a central photon energy of 2.2 eV. For the probe we employed equally short pulses centred around 1.3 eV and with a polarization perpendicular to that of the pump.

**Figure 1:**
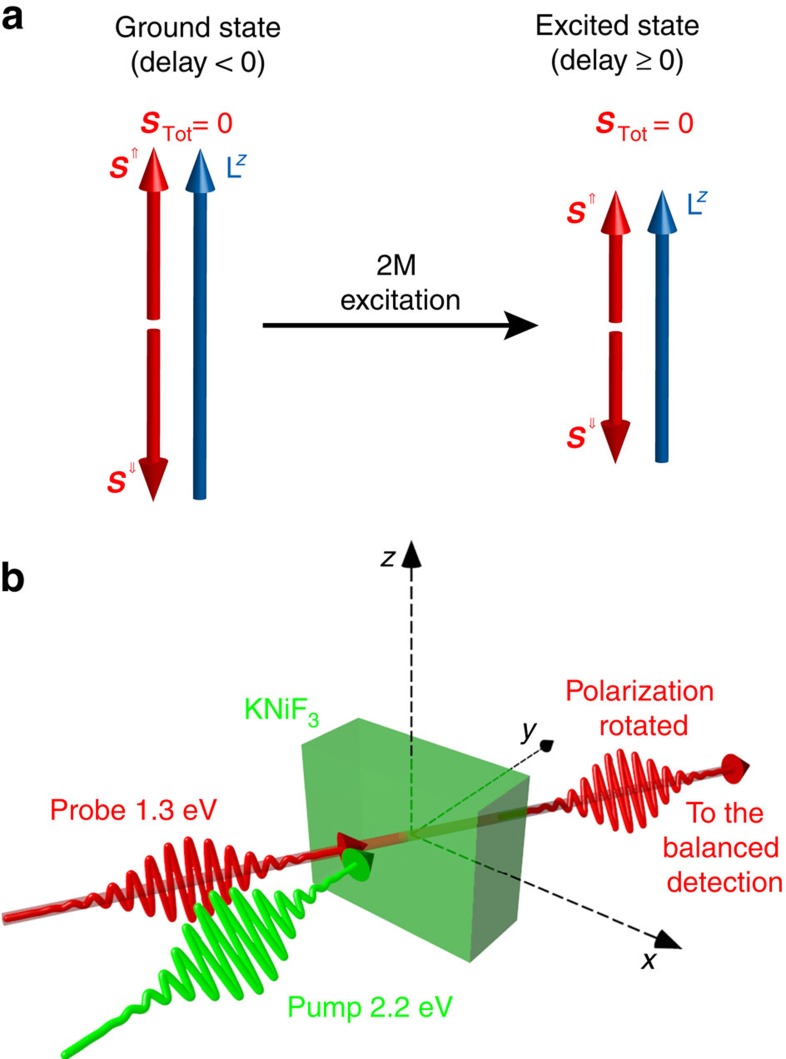
2M mode and experimental configuration. (**a**) The 2M excitation is equivalent to a spin-flip event per sublattice. Thus, the magnetization of each sublattice (***S***^⇑^ and ***S***^⇓^, represented by the two red arrows with opposite orientation) and, therefore, the antiferromagnetic vector (L^*z*^, blue arrow) is decreased in the excited state. The sum of the spins of the two sublattices, thus the total magnetization, vanishes both in the ground and in the excited state. (**b**) Schematic representation of the experimental geometry. The pump (green pulse) photon energy was tuned to 2.2 eV in the transparency window of the material. This choice avoided contributions of laser-heated electrons and phonons to the spin dynamics^19^. The central photon energy of the probe beam (red pulse) was 1.3 eV. The arrows indicates the direction of propagation of the two laser beams.

Light scattering by a magnon pair can be visualized as two spin-flip events, one on each sublattice, such that the total spin remains unchanged^6^,^7^,^14^ (Fig. 1a). Consequently, the transient magneto-optical Faraday and Kerr effects, which measure light-induced variations of the total spin, fail to track the dynamics of such a magnetic excitation. On the other hand, the 2M process is expected to be revealed by those second-order magneto-optical effects, which depend quadratically on the transversal spin components (that is, on the spin deviations), so that a pair of spin-flip events can be detected even if the total spin is unaffected (see discussion in Method section and equations (6) and (7)). Such a magneto-optical effect depends on the spin operators via the same spin-correlation function appearing in the Heisenberg term of the Hamiltonian^17^ (equation (9)). In particular, we define the antiferromagnetic linear dichroism (ALD) in terms of the symmetric components of the dielectric tensor 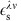 for which 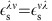, where *ν* and *λ* are indices. The following definition holds:^17^,^18^


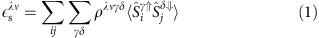


where *ρ*^*λνγδ*^ is a magneto-optical polar fourth rank tensor and *λνγδ* are spatial coordinate indices, while 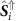 and 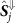 are the spin operators located on two nearest-neighbour sites (*i*, *j*), belonging to oppositely oriented magnetic sublattices (⇑ and ⇓). Note that the ALD induces a rotation of the probe polarization in the experimental configuration shown in Fig. 1b (see Methods section). It is crucial to notice that the two spins in the correlation function belong to two different ionic sites on opposite sublattices. This fact differentiates the ALD from the more conventional magnetic linear birefringence and dichroism (see equation (9) and discussion in the Methods). The macroscopic magnetic order of an ideal Heisenberg antiferromagnet is conveniently described in terms of the antiferromagnetic vector **L**, which is the order parameter^18^ and is defined as


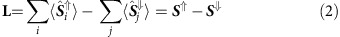


where ***S***^⇑^ and ***S***^⇓^ are the total spins of the two sublattices (Fig. 1a). Our considerations about the dynamics of the spin system triggered by the impulsive excitation of the 2M mode are based on the approximation of non-interacting magnons. In this framework it is straightforward to demonstrate that the *z*-projection of **L** has the same time dependence as the spin-correlation function, at the leading terms in the magnon operators (equations (11) and (12)). Here *z* is the direction parallel to the spins in equilibrium. The dynamics of L^*z*^, at the leading order in the excitation intensity, is given by


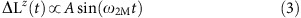


where *ω*_2M_ is the frequency of the 2M mode and *A* is the amplitude. Equation (3) describes a purely longitudinal, non-precessional, dynamics of the antiferromagnetic vector (see Supplementary Note 2 for the complete derivation). Therefore, by detecting the femtosecond dynamics of the ALD, we access the time evolution of the macroscopic order parameter via the spin-correlation function, which ultimately defines also the dynamics of the exchange energy.

### Femtosecond dynamics of the magnetic system

**Figure 2:**
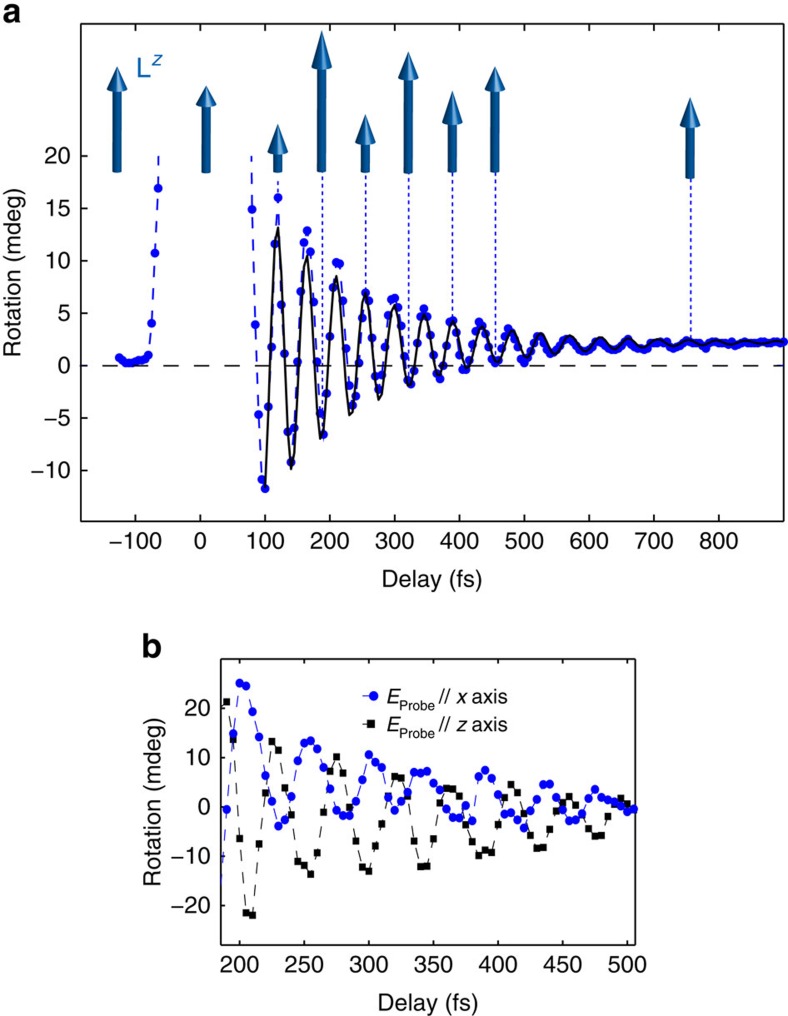
Laser-induced dynamics of the antiferromagnetic vector. (**a**) The transient rotation of the probe polarization was measured with the electric fields of the pump and the probe beams linearly polarized along the *z* and *x* axes, respectively. The pump fluence was set to ≈8.6 mJ cm^−2^. The corresponding dynamics of L^*z*^ (blue arrows) is schematically represented. When the pump pulses impinge on the sample (0 delay) L^*z*^ decreases, due to the generation of magnons. At positive delays, oscillations at the frequency of the 2M mode are visible (equation (3)). The black line is a fit to the data (equation (13)). (**b**) The phase of the oscillation is shifted by *π* when the orientation of the electric field of the probe (***E***_Probe_) is rotated by 90°. The pump beam was linearly polarized along the *z* axis, the fluence was set to ≈12 mJ cm^−2^. The measurements reported in both panels were performed at 80 K.

**Figure 3:**
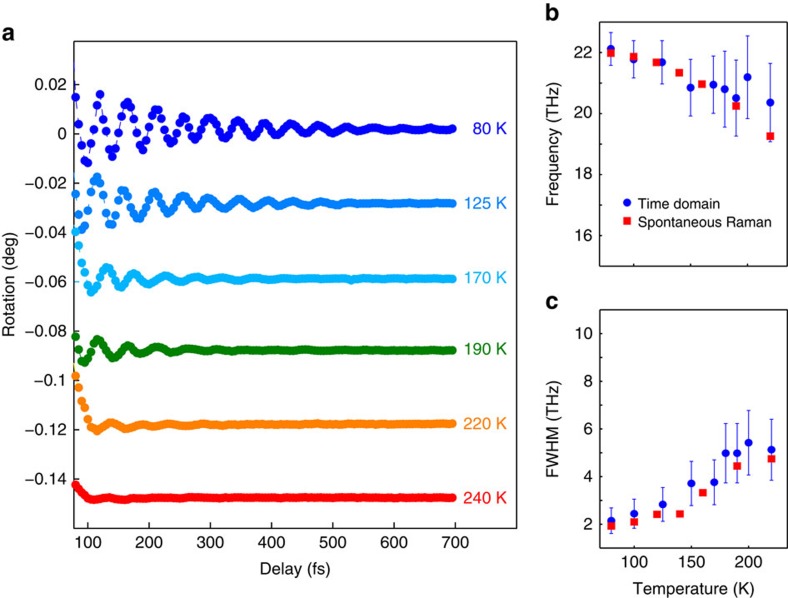
Temperature dependence of the ultrafast spin dynamics. (**a**) Rotation of the probe polarization as a function of the temperature. The pump and the probe beams are linearly polarized along the *z* and *y* axes, respectively. The fluence is ≈8.6 mJ cm^−2^. (**b**,**c**) The temperature dependence of the frequency and linewidth of the 2M Raman peak (red squares) compared with the trends obtained by applying the Fourier transform to the time-domain data (blue circles). The error bars in **b** and **c** for the time-domain data are defined as the half width at half maximum of the 2M frequency distribution. The error bars for the Raman data are defined by the instrumental sensitivity (≈2 cm^−1^) and are not visible in this scale.

Figure 2a shows the typical result of a time-resolved measurement of the laser-induced spin dynamics. The transient rotation of the probe polarization shows oscillations in time with a period of ≈45 fs (that is, a frequency of ≈22 THz) that are damped on a 500-fs timescale. The oscillatory dynamics is superimposed on an incoherent increase of the background, as it is clear from the difference between the time trace and the zero line at longer delays (>500 fs). The spectrum of the oscillation measured in the time-domain experiment closely matches the 2M mode as measured by spontaneous Raman. To definitely assess the nature of the 22 THz mode, we performed temperature-dependent measurements (Fig. 3a) and we compared the temperature dependence of the time-domain signal with that of the spontaneous Raman spectra of the 2M bound state (Fig. 3b,c). Figure 3 shows that the frequency and the lifetime of the pump-induced oscillations decrease as the Néel point is approached, in qualitative and quantitative agreement with spontaneous Raman data^6^,^14^. Thus, Fig. 2a unambiguosly reveals the femtosecond spin dynamics triggered by the impulsive excitation of the 2M mode in KNiF_3_, which is not accessible with any other experimental approach.

A fit to the data in Fig. 2a (see Methods section, equation (13)) gave *τ*_d_=(167±4) fs (damping of the coherent oscillations) and *τ*_r_=(255±16) fs (rise time of the incoherent background response). While *τ*_d_ represents the decoherence of the 2M band, we interpret *τ*_r_ as the characteristic demagnetization time of the two sublattices, solely driven by magnetic interactions^19^. As a laser pulse excites a continuum of magnons with different frequencies, the damping time *τ*_d_ of the oscillations observed in our experiment is actually due to the decoherence of the inhomogeneous ensemble of the coherently excited magnons. This is usually described^20^ by means of the characteristic time 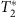. The demagnetization of the sublattices is a result of the heating of spins, which is caused by the decoherence of single-magnon modes^19^ in the ensemble, on a timescale generally indicated with *T*_2_ (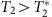)^20^. Measuring the probe polarization dependence of the signal (Fig. 2b) allowed us to demonstrate that the detected rotation of the polarization in our experiment arises from the symmetric components of the 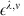 tensor and, in particular, from the ALD. In fact the change of sign and the different amplitude of the two data sets shown in Fig. 2b is predicted by the microscopic theory of the ALD (Supplementary Notes 2 and 3).

### Coherent control

**Figure 4:**
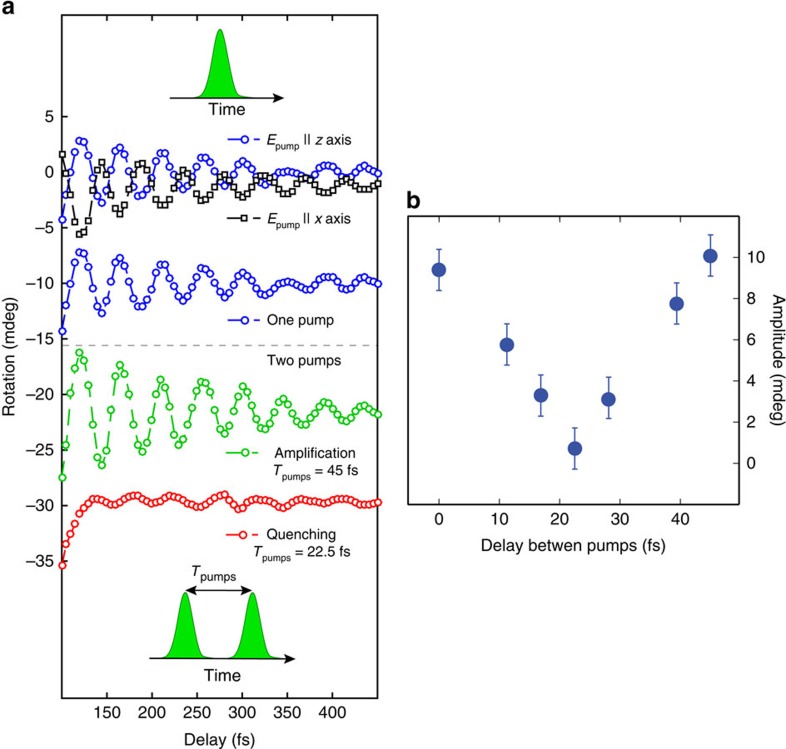
Coherent control of the spin dynamics. (**a**) The blue and black dotted traces were obtained using a single-pump beam linearly polarized along the *z* axis (blue circles) and the *x* axis (black squares). The phase of the oscillations is shifted by *π*. The single-pump measurement is repeated for the sake of comparison with the results of the double-pump experiments. The green-circle data set was observed by delaying two pump pulses by *T*_pumps_=45 fs, that is, the period of the 2M mode. The coherent amplification of the signal is observed. The coherent oscillations were quenched, when the delay between the two pump pulses was set to *T*_pumps_=22.5 fs. The fluence of both pump beams was set to ≈1.5 mJ cm^−2^. The temperature was 80 K. The probe beam was polarized along the *x* axis. (**b**) Amplitude of the first oscillation as a function of the delay between the two pumps (*T*_pumps_), the periodic trend is clear. The error bars are defined as twice the sensitivity of our set-up.

The ability to arbitrarily manipulate the properties of spin waves is a necessary requirement for the development of magnon-based devices^21^. Figure 4a demonstrates the control of the phase of the nanomagnons. The upmost time traces were measured exciting the sample with orthogonally polarized pump beams. A clear *π* shift of the phase of the oscillations is observed, if the polarization of the excitation beam is rotated by 90°. This phase shift is due to the non-equivalence of the excitations of the magnetic system induced by light linearly polarized along and orthogonally to the direction of the spins. This interpretation is substantiated by a symmetry analysis of the two-magnon excitation (Supplementary Note 4), based on the point group of KNiF_3_. The coherent nature of the photo-induced spin dynamics enables the manipulation of the amplitude of the spin waves, by means of a sequence of laser pulses. We demonstrate this effect in Fig. 4a employing two time-delayed excitation pulses. Tuning the delay we can either amplify or strongly quench the oscillations compared with the case when the spin dynamics is triggered by a single-pump pulse. Figure 4b reveals the periodic dependence of the amplitude of the first magnetic oscillation on the delay between the pump pulses. Such a coherent control of the femto-nanomagnons allows a direct and complete manipulation of the antiferromagnetic order parameter on the femtosecond timescale.

### Time-frequency analysis

**Figure 5:**
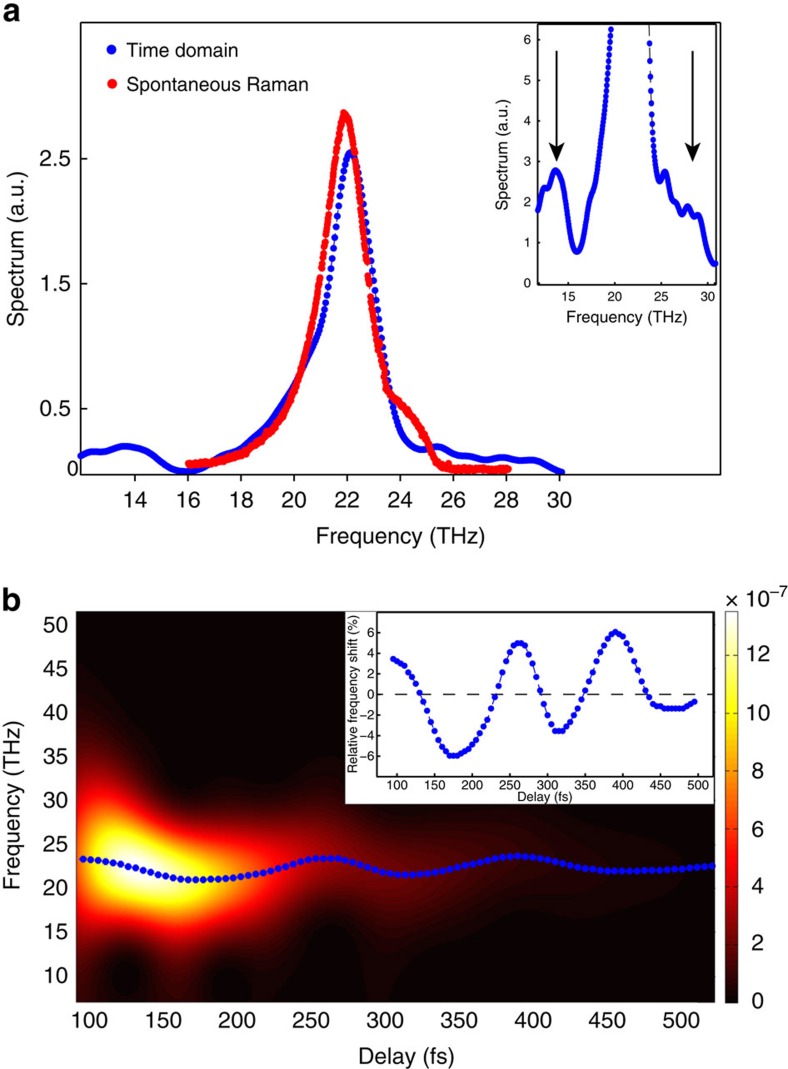
Spectrum of the ultrafast response of the antiferromagnetic vector. (**a**) The Fourier transform of the time trace measured at 80 K (blue curve) is compared with the spontaneous Raman spectrum obtained at the same temperature (red curve). In the inset a zoom of the Fourier transform reveals two sidebands at ≈±7.5 THz away from the peak frequency. (**b**) The squared modulus of the Wigner distribution of the signal is represented by the colour plot. At each time step a blue dotted line highlights the maximum of the spectrum, in which black represents zero intensity. In the inset the relative frequency shift (equation (4)) is plotted as a function of the delay showing ≈7.5 THz oscillations.

The real-time measurement of the ultrafast spin dynamics triggered by short-range magnons allows us to disclose another phenomenon not observable with equilibirum techniques. Figure 5a shows the spectrum of the time trace in Fig. 2a obtained via a Fourier transform (blue curve). On the same graph we plot the spectrum of the 2M mode measured via spontaneous Raman scattering at the same temperature (red curve). The zoom in the inset of Fig. 5a shows two sidebands at about 7.5 THz from the central 2M frequency, which appear to originate from a modulation of the 2M mode in the time domain. Figure 5b shows a two-dimensional spectrogram obtained by performing a time-frequency analysis^22^,^23^ of the data in Fig. 2a (Supplementary Note 5). The colour map in Fig. 5b represents the time-dependent spectrum of the 2M mode. The frequencies of the peak of the spectrum at different time delays are traced by a blue dotted line. This curve displays a periodic oscillation of *ν*_2M_, which is consistent with the observation of the sidebands in the inset of Fig. 5a. We define the relative frequency shift as


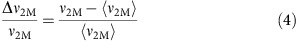


where 〈*ν*_2M_〉 is the average frequency in the temporal interval where the oscillations have a significant amplitude (0–500 fs). We plot the peaks of the spectra obtained by the time-frequency analysis as a function of the delay in the inset of Fig. 5b. The frequency of the modulation of *ν*_2M_ is ≈7.5 THz, which corresponds to the frequency of the infrared-active phonon^24^,^25^ (≈7.7 THz), assigned to the stretching vibration of the Ni–F–Ni bond. Unlike other phonon modes in this material, the frequency of the stretching mode is temperature-independent^24^,^25^, which is consistent with the data (Supplementary Fig. 2). Considering only the term of the polarization of the first order in the electric field of light, it would be concluded that this stretching mode is not Raman-active^26^. However, the light–matter interaction at the next order (hyper-Raman scattering^26^,^27^) allows to excite lattice vibrations with the symmetry of the stretching mode (*F*_1*u*_) in cubic crystals^27^. It is well established that a frequency modulation as the one shown in Fig. 5b can be modelled in terms of coupled oscillators^23^. Therefore, we assign the modulation of *ν*_2M_ to the interaction on the femtosecond timescale between the stretching mode and the 2M mode, which are simultaneously and coherently excited by the laser pulse. The 2M–phonon interaction was previously suggested^11^, however our time-resolved experiment provides the first evidence of this effect.

## Discussion

We would like to underline that the dynamics here reported is totally different from the spin response previously observed after the photo-excitation of the low-energy magnons with wavevectors at the centre of the Brillouin zone in this material^19^. In fact, our previous investigation revealed spin oscillations with the period of 11 ps (that is, a 90-GHz frequency), which are still visible 200 ps after the photo-excitation. This spin dynamics was generated by a single spin-flip event (that is, one-magnon mode), thus the magnetic linear birefringence, which depends quadratically on the spin of a single atom but only linearly on the spin deviation (equation (9)), was employed to probe the response of the magnetic system. The interaction relevant for the low-energy magnons is the spin–orbit coupling, which defines the frequency of such collective excitations^6^. The use of 100-fs laser pulses prevented the access to high-frequency magnetic modes^19^. The present work describes a completely different regime of spin dynamics, whose characteristic timescale is determined by the exchange energy^6^. Moreover, the measurements of the time evolution of the spin-correlation function (via ALD) provide an access to the dynamics of the exchange energy. Within the approximations of our model, the spin-correlation dynamics corresponds to the longitudinal dynamics of the order parameter (equations (11) and (12) and Supplementary Note 2).

In summary, our results reveal the dynamics of the macroscopic order parameter triggered by impulsively excited nanometre-wavelength and femtosecond-period magnons. Moreover, our experiment pushes the coherent control of magnons, previously demonstrated at the centre of the Brillouin zone^28^,^29^, to the edges of the Brillouin zone, laying the foundations for a magnon-based nanotechnology operating in the 20-THz regime. Our investigation concerned an ideal Heisenberg antiferromagnet, however the approach here employed to study the femtosecond dynamics of the macroscopic magnetic order parameter caused by short-range spin excitations can be employed in a broad group of multisublattice systems^6^,^7^. In particular, it will become possible to monitor the evolution of the exchange energy during a photo-induced phase transition and to probe the femtosecond dynamics of the sub-nanometre range spin correlations in strongly correlated materials. This might even elucidate the dynamical interplay between short-range spin excitations and high-temperature superconductivity in cuprates^30^,^31^,^32^.

## Methods

### Sample

Our sample was a 340-μm-thick (100) plane-parallel plate of KNiF_3_, which has a perovskite crystal structure (point group *m*3*m*). Two equivalent Ni^2+^ sublattices are antiferromagnetically coupled below the Néel temperature *T*_N_=246 K (ref. 19). This material is known to be a cubic Heisenberg antiferromagnet because of its very weak anisotropy. The positive sign of the cubic magnetic anisotropy constant determines the alignment of spins along the [001], [010] or [100] axes^18^. The measurements on the KNiF_3_ sample are carried out at a minimum temperature of 77 K in a liquid nitrogen cryostat. The temperature of the sample is monitored by a thermocouple placed on the sample holder.

### Light source

For the pump–probe experiments we used a regeneratively amplified mode-locked Ti:Sapphire laser, providing 150-fs, 500-μJ pulses at 780 nm and 1 kHz repetition rate. The laser drives two non-collinear optical parametric amplifiers (NOPAs) operating in two different spectral ranges^33^. Both NOPAs are pumped by the second harmonic of the laser (that is, 390 nm) and seeded by the white-light continuum produced by focusing the 780-nm beam into a sapphire plate. The amplified pulse from the first NOPA, which initiates the dynamics (pump), has a spectrum spanning the 500–700 nm range and is compressed to nearly transform-limited duration (that is, 8 fs) by a pair of custom-made chirped mirrors. The amplified pulse, generated by the second NOPA (probe), covers the frequency range between 820 and 1050 nm, and is compressed to nearly transform-limited duration (that is, 13 fs) by a couple of fused silica prisms. The temporal resolution of the set-up has been characterized by the cross-correlation frequency-resolved optical gating technique and was below 20 fs (ref. 32). The pump and probe beams were focused on the sample by a spherical mirror down to approximately 100 and 70 μm spot sizes, respectively. The high temporal resolution is preserved by using a very thin (200 μm) fused silica window as optical access to the cryostat. The two pump pulses employed to show the coherent control of the 2M mode were generated in a Michelson interferometer scheme. One of these beams was reflected by mirrors mounted on a stepper-motor delay line, able to introduce a minimum time delay of 0.3 fs. The two orthogonal polarizations were obtained with a polarizer sheet (thickness ≈0.3 mm), to avoid the use of thicker waveplates and the consequent broadening of the pulse duration.

### Detection scheme

We measured the pump-induced rotation of the probe polarization employing a balanced-detection scheme. The transmitted probe is split by a Wollaston prism into two orthogonal linearly polarized beams, and focused on a couple of balanced photodiodes. The Wollaston prism is rotated to equalize the probe intensities on the two photodiodes. The pump-induced imbalance of the signal registered by the two photodiodes is measured by a lock-in amplifier, which is locked to the modulation frequency of the pump beam (that is, 500 Hz). Our apparatus was able to detect rotations of the polarization on the order of 1 mdeg.

### Excitation mechanism

As explained in the main text, a light-induced modification of the exchange interaction generates the 2M mode^6^,^7^. This concept can be easily verified by taking into account the transient value of the exchange in the energy of the magnetic medium


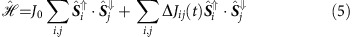


where Δ*J*_*ij*_(*t*) is the laser-induced modification of the exchange interaction, while *J*_0_ is the value of the exchange in the ground state. The *ij* indices refer to the anisotropic coupling of light with the magnetic system, determined by the polarization of the laser beam, which breaks the isotropic symmetry of *J*_0_ (Supplementary Note 2). The second term of the equation can be rewritten by expanding the dot product in the following way


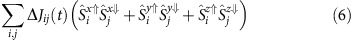


Defining *z* as the direction along which the spins are oriented at equilibrium, this equation shows that the modification of the exchange generates terms in the energy quadratic in the transversal spin components (that is, *x* and *y*). Such contributions describe the process of generation of two magnons^6^ reported in Fig. 1a. This becomes evident by introducing the conventional ladder operators 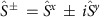, in terms of which we can rewrite equation (6) as


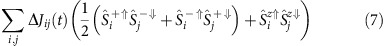


The physical interpretation of the first two terms of the last equation consists in two spin-flip processes, one on each sublattice. Once a positive direction of the quantization axis is chosen (direction of L^*z*^ in Fig. 1a), flipping a spin on the ⇑(⇓) sublattice results in a decrease(increase) of the total spin, taken into account by 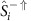
(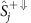). In conclusion, a transient modification of the exchange interaction, which breaks the isotropic symmetry of *J*_0_, triggers two magnons described by the quadratic dependence of the Hamiltonian on the transversal spin components.

### Antiferromagnetic linear dichroism

The Hamiltonian representing the interaction between light and the magnetic system can be written as^6^


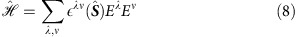


where 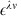 is the dielectric tensor that depends on the spin (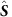), while *λ*, *ν* are indexes and *E* is the electric field of light. The tensor 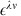 can be expanded in powers of the spin operators. Following a well-established approach^6^,^17^, we may express the spin-dependent components of 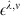 as


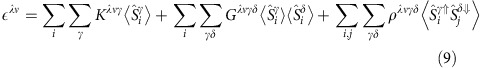


where higher-order terms are omitted. Each term in this expansion represents a magneto-optical effect and a Raman mode, given the intimate connection between the tensors defining the Raman scattering on magnons and the magneto-optical coefficients^6^,^34^. The first and second terms involve spin operators at a single ionic site *i*, they describe the linear magneto-optical effects: the complex Faraday effect and the magnetic linear birefringence(and dichroism), respectively. In terms of Raman scattering the first two terms of equation (9) are connected with the lowest energy magnetic excitations, correspondent to a single spin-flip process, the so called one-magnon modes. These spin waves have wavevectors close to the centre of the Brillouin zone and their frequencies are mostly defined by the spin–orbit coupling. Note that although the second term is quadratic in the spin, it describes a single-magnon excitation and a linear magneto-optical effect. In fact this term is linear in the spin deviation, that is, in the spin transversal component, which implies a linear dependence on the spin ladder operators and therefore the generation of a single magnon^6^ (see discussion in the previous section). The magnetic linear birefringence was employed to probe the spin dynamics in reference^19^, which accordingly reports the excitation and the time evolution of the low-energy one-magnon mode in KNiF_3_.

In the present study we measure the ALD, which is accounted for by the last term in equation (9): the quadratic dependence on the spin deviations (equations (6) and (7)) of this term allows to unravel the dynamics of the 2M mode, triggered by two spin-flip processes. Moreover, the two spin operators in this term belong to different sublattices, being placed on different ionic sites *i*, *j*. This is the pivotal difference between this magneto-optical effect and the others described so far: the ALD probes the correlations between spins on different sites. Since in KNiF_3_ only the nearest-neighbour interaction is significant, the dynamics of the spin-correlation function in the last term of equation (9) defines also the dynamics of the exchange energy. The transient ALD, which consists of a different absorption for light beams linearly polarized along and orthogonally to the direction of the antiferromagnetic vector, results in the detected rotation of the probe polarization.

### Dynamics of the order parameter

The dynamics of the spin-correlation function can be related to the dynamics of the order parameter in a straightforward way. The *z*-component of the local spins on sites *i*, *j*, belonging to the ⇑ and ⇓ sublattices respectively, can be written as


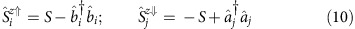


where the operators 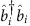 and 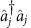 (introduced rigorously in Supplementary Note 2) represent the number of spin deviations from the maximum values of local spins for both sublattices ±*S*. We can then write the expectation value of the spin-correlation function on a time-dependent state |*ψ*(*t*)〉 as


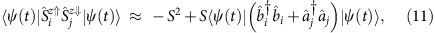


where only the leading terms in the creation and destruction operators have been considered. Recalling the definition of the antiferromagnetic vector in equation (2), the time-dependent *z*-component of the order parameter is given by


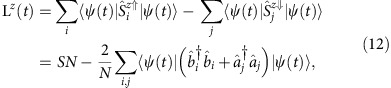


where *N* is the total number of spins. It stems from equations (11) and (12) that the time-dependent parts of the spin-correlation function and of the antiferromagnetic vector are proportional.

### Fitting procedure

The transient rotation of the probe polarization was fitted employing the following function:


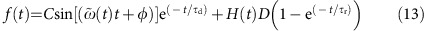


where *C* and *D* are amplitude coefficients, *φ* is the phase of the oscillations, *H*(*t*) is the Heaviside function, *τ*_d_ is the damping time of the oscillations and *τ*_r_ is the characteristic rise time of the incoherent contribution to the signal. Considering the outcome of the Wigner analysis in Fig. 5b, we employed a time-dependent frequency 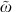 in the sinusoidal function, namely


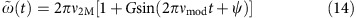


where *ν*_2M_ is the 2M frequency, *G* is an amplitude coefficient, *ν*_mod_ is the modulation frequency and *ψ* is the phase. From the Wigner analysis we set the following parameters: *ν*_2M_=22.12 THz; *G*=0.002; *ν*_mod_=7.5 THz; *ψ*=45°; and *D*=2.3·10^−3^ deg. The fit parameters allowing to reproduction at best the data (Fig. 2) are as follows: *C*=(2.5±0.1)·10^−2^ deg; *φ*=(220±1)°; *τ*_d_=(167±4) fs; and *τ*_r_=(255±16) fs.

## Additional information

**How to cite this article**: Bossini, D. *et al*. Macrospin dynamics in antiferromagnets triggered by sub-20 femtosecond injection of nanomagnons. *Nat. Commun.* 7:10645 doi: 10.1038/ncomms10645 (2016).

## Supplementary Material

Supplementary Information Supplementary Figures 1-2, Supplementary Notes 1-5 and Supplementary References
