# Supplementary material for: Macrospin dynamics in antiferromagnets triggered by sub-20 femtosecond injection of nanomagnons
Source: Nat Commun. 2016 Feb 5;7:10645. doi: 10.1038/ncomms10645 (PMC4748265; doi:10.1038/ncomms10645)
Supplement: Supplementary Information — Supplementary Figures 1-2, Supplementary Notes 1-5 and Supplementary References [file ncomms10645-s1.pdf]

Supplementary Figures

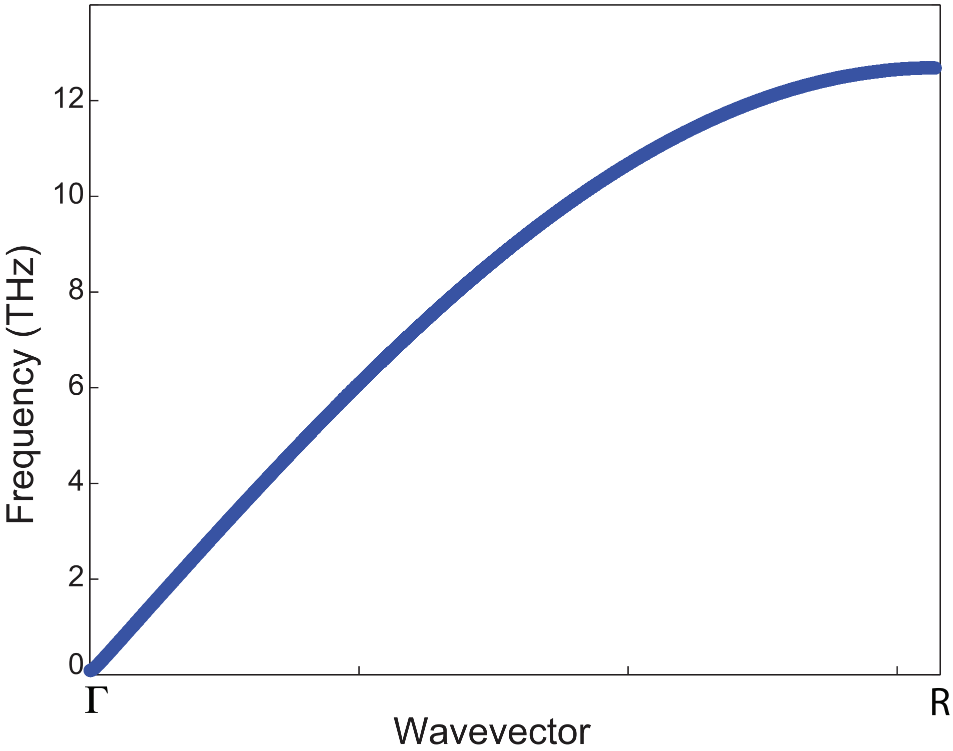

Supplementary Figure 1. **Magnon dispersion in $KNiF_3$.** The dispersion is approximately linear for small wave vectors, while it tends to flatten at the zone edges. At the R point the frequency of the magnons is $\omega_R \approx 12$ THz, in agreement with the data presented in the main text, $\omega_{2M} = 22$ THz $\approx 2\omega_R$ within the correction due to magnon-magnon interaction.

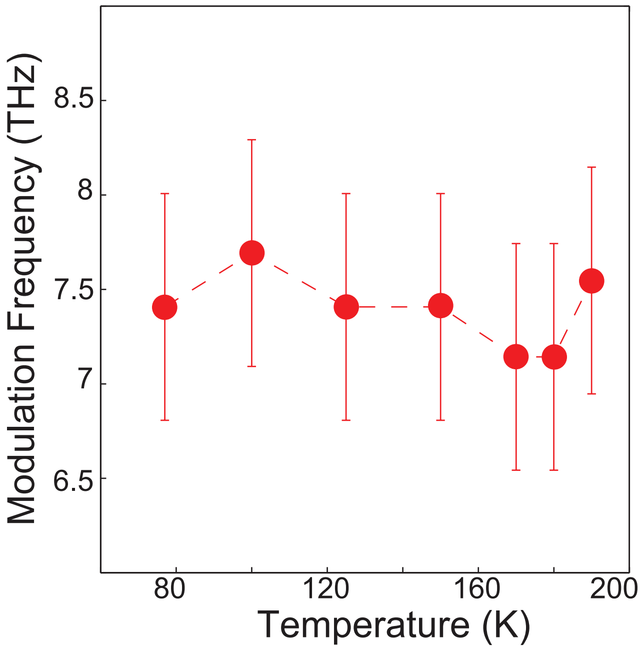

Supplementary Figure 2. **Temperature dependence of the modulation frequency.** The modulation frequency is obtained from the Wigner analysis of the time traces measured at different values of temperature. The error bars are defined from the uncertainty in determining the positions of the maxima (i.e. the experimental delay-time step) of the oscillating trends of the 2M frequency.

Supplementary notes

Supplementary Note 1

Our experiment is performed on the cubic Heisenberg antiferromagnet $KNiF_3$, which can be described in equilibrium by the following Hamiltonian:

$$\hat{H} = J \sum_{\langle i,j \rangle} \hat{\boldsymbol{S}}_i^{\Uparrow} \cdot \hat{\boldsymbol{S}}_j^{\Downarrow}, + g\mu_B H_A \left(\sum_i \hat{S}_i^{z\Uparrow} - \sum_j \hat{S}_j^{z\Downarrow} \right), \tag{1}$$

where $J > 0$ is the exchange parameter, the sum runs over nearest-neighbour pairs $\langle i, j \rangle$, and $\hat{\boldsymbol{S}}_i^{\Uparrow}$, $\hat{\boldsymbol{S}}_j^{\Downarrow}$ are the spin operators related to the magnetic sites i and j, belonging, respectively, to the "spin-up" sublattice $\Uparrow$ and the "spin-down" sublattice $\Downarrow$. The anisotropy is expressed by an effective field H_A (multiplied by the g-factor g and the Bohr magneton μ_B) which aligns the spins of the two sublattices along the z direction. The resulting dispersion relation of magnons [1] is

$$\omega_{\boldsymbol{q}} = \sqrt{(\omega_E + \omega_A)^2 - (\omega_E \gamma_{\boldsymbol{q}})^2}, \tag{2}$$

where $\omega_E \equiv JSz$, z is the number of nearest neighbours ($z = 6$ for the cubic crystal that we are considering here), $\omega_A \equiv g\mu_B H_A$, and $\gamma_{\boldsymbol{q}}$ is given by

$$\gamma_{\boldsymbol{q}} = \frac{1}{z} \sum_{\boldsymbol{\delta}} \mathrm{e}^{\mathrm{i}\boldsymbol{q}\cdot\boldsymbol{\delta}}, \tag{3}$$

where the z vectors $\{\boldsymbol{\delta}\}$ connect a site on the $\Uparrow$ sublattice to its z nearest neighbours on the $\Downarrow$ sublattice. For the cubic crystal, $\{\boldsymbol{\delta}\} = \{a(\pm 1, 0, 0), a(0, \pm 1, 0), a(0, 0, \pm 1)\}$, where a is the lattice spacing, therefore $\gamma_{\boldsymbol{q}}$ becomes

$$\gamma_{\boldsymbol{q}} = \frac{1}{3}[\cos(q_x a) + \cos(q_y a) + \cos(q_z a)] = \gamma_{-\boldsymbol{q}}. \tag{4}$$

The *magnetic* Bravais lattice coincides with one of the two sublattices ($\Uparrow$ or $\Downarrow$), and it is a FCC lattice with cubic edge length equal to $2a$. All the $\boldsymbol{q}$ points in the magnetic first Brillouin zone satisfy $|q_\alpha| \leq \pi/(2a)$, for $\alpha = x, y, z$; therefore, $0 \leq \gamma_{\boldsymbol{q}} \leq 1$ over the whole first Brillouin zone. In Supplementary Fig. 1 we plot the energy dispersion of magnons in $KNiF_3$ evaluated along the $\overline{\Gamma \mathrm{R}}$ direction, computed using Supplementary Eq.(2), where

$\Gamma \equiv (0,0,0)$ and $\mathrm{R} \equiv \frac{\pi}{2a}(1,1,1)$ are, respectively, the center and one of the corners of the first Brillouin zone. The effective fields and the g factor values were taken from Ref.[2].

Supplementary Note 2

Within the framework of an analytically solvable model, we now determine the time-dependent spin correlation functions, which are responsible for the modulation of the dielectric tensor.

For simplicity, we neglect the anisotropy field, so the equilibrium Hamiltonian is given by Supplementary Eq.(1) with $H_A = 0$. It can be represented in terms of magnon excitations on the top of the Néel state by means of the standard Holstein-Primakoff expansion [3, 4]. The free-magnon Hamiltonian is

$$\hat{H}_0 \equiv \sum_{\boldsymbol{q}} \omega_{\boldsymbol{q}} \left(\hat{\alpha}^\dagger_{\boldsymbol{q}} \hat{\alpha}_{\boldsymbol{q}} + \hat{\beta}^\dagger_{\boldsymbol{q}} \hat{\beta}_{\boldsymbol{q}} \right), \tag{5}$$

where $\omega_{\boldsymbol{q}} = JSz\sqrt{1-\gamma_{\boldsymbol{q}}^2}$, the $\boldsymbol{q}$ vectors run over the *magnetic* first Brillouin zone, and the two species of independent magnon fields for an antiferromagnet are denoted as $\hat{\alpha}$ and $\hat{\beta}$. They are obtained from the following Bogoliubov transformation:

$$\begin{aligned}
&\hat{a}_{\boldsymbol{q}} \equiv u_{\boldsymbol{q}} \hat{\alpha}_{\boldsymbol{q}} + v_{\boldsymbol{q}} \hat{\beta}^\dagger_{-\boldsymbol{q}}, \qquad \hat{b}_{\boldsymbol{q}} \equiv u_{\boldsymbol{q}} \hat{\beta}_{\boldsymbol{q}} + v_{\boldsymbol{q}} \hat{\alpha}^\dagger_{-\boldsymbol{q}}, \\
&u_{\boldsymbol{q}} = \left(\frac{1}{2\sqrt{1-\gamma_{\boldsymbol{q}}^2}} + \frac{1}{2} \right)^{1/2}, \quad v_{\boldsymbol{q}} = -\left(\frac{1}{2\sqrt{1-\gamma_{\boldsymbol{q}}^2}} - \frac{1}{2} \right)^{1/2},
\end{aligned} \tag{6}$$

where $\hat{a}_{\boldsymbol{q}}$ and $\hat{b}_{\boldsymbol{q}}$ are the Fourier transforms of the Holstein-Primakoff bosons, which measure the deviations of the z components of the local spins from their values in the Néel state (we are using the convention for which $\hat{S}_i^{z\Uparrow} \equiv S - \hat{b}_i^\dagger \hat{b}_i$ and $\hat{S}_j^{z\Downarrow} \equiv -S + \hat{a}_j^\dagger \hat{a}_j$). Neglecting all the interaction terms between magnons, we use $\hat{H}_0$ as given in Supplementary Eq.(5) as the model Hamiltonian for our magnetic system in equilibrium.

Interaction between light and the magnetic system

The Hamiltonian describing the magnetic system in the pump-probe experiment is:

$$\hat{H}(t) \equiv \hat{H}_0 + \hat{H}_1(t) + \hat{H}_2(t-\tau), \tag{7}$$

where $\hat{H}_1(t)$ and $\hat{H}_2(t-\tau)$ describe the time-dependent perturbations induced in the magnetic system, respectively, by the pump and the probe laser fields. The delay between the two pulses is denoted as τ.

We model the coupling between light and the spin system analogously to Ref.[5]. Assuming that the pump excitation is instantaneous, we write

$$\hat{H}_1(t) \approx \delta(t)\frac{4\pi I_1}{n_R c}\sum_{\langle i,j\rangle}\Xi_{ij}\left(\frac{\hat{S}_i^{+\Uparrow}\hat{S}_j^{-\Downarrow}+\hat{S}_i^{-\Uparrow}\hat{S}_j^{+\Downarrow}}{2}+A\hat{S}_i^{z\Uparrow}\hat{S}_j^{z\Downarrow}\right), \tag{8}$$

where A is an anisotropy parameter ($A=1$ in the isotropic case), I_1 is the integrated intensity of the pump pulse, n_R is the refractive index and c is the speed of light. The parameter Ξ_{ij} depends on the polarizations and wave vectors of incident and scattered light. We assume [5] that it depends on i and j only via $\boldsymbol{R}_i-\boldsymbol{R}_j\equiv\boldsymbol{\delta}$, so that

$$\Xi_{ij}\equiv\Xi(\boldsymbol{\delta})=\frac{2}{N}\sum_{\boldsymbol{q}}\mathrm{e}^{\mathrm{i}\boldsymbol{q}\cdot\boldsymbol{\delta}}\Xi_{\boldsymbol{q}}, \qquad \Xi_{\boldsymbol{q}}=\sum_{\boldsymbol{\delta}}\mathrm{e}^{\mathrm{i}\boldsymbol{q}\cdot\boldsymbol{\delta}}\Xi(\boldsymbol{\delta})=\Xi_{-\boldsymbol{q}}. \tag{9}$$

The Hamiltonian given in Supplementary Eq.(8) has the form of a time-dependent exchange interaction. In the isotropic case ($A=1$), one recovers Eq.(5) of the main text, with $\Delta J_{ij}(t)=\delta(t)\,\Xi_{ij}\,4\pi I_1/(n_R c)$. For $A\neq 1$, the time-dependent exchange is anisotropic.

After re-writing Supplementary Eq.(8) in terms of the magnon operators and retaining only quadratic terms, we obtain

$$\hat{H}_1(t)\equiv\delta(t)\hat{F}, \tag{10}$$

where the dimensionless operator $\hat{F}$ has the form

$$\hat{F}\equiv F_0+\sum_{\boldsymbol{q}}f'_{\boldsymbol{q}}\left(\hat{\alpha}^\dagger_{\boldsymbol{q}}\hat{\alpha}_{\boldsymbol{q}}+\hat{\beta}^\dagger_{\boldsymbol{q}}\hat{\beta}_{\boldsymbol{q}}\right)+\sum_{\boldsymbol{q}}f_{\boldsymbol{q}}\left(\hat{\alpha}^\dagger_{\boldsymbol{q}}\hat{\beta}^\dagger_{-\boldsymbol{q}}+\hat{\alpha}_{\boldsymbol{q}}\hat{\beta}_{-\boldsymbol{q}}\right)\equiv F_0+\hat{F}_1, \tag{11}$$

where

$$\begin{aligned} f_{\boldsymbol{q}} &\equiv \frac{4\pi I_1}{n_R c}S\,\frac{\Xi_{\boldsymbol{q}}-A\gamma_{\boldsymbol{q}}\Xi_{\boldsymbol{0}}}{\sqrt{1-\gamma_{\boldsymbol{q}}^2}},\\ f'_{\boldsymbol{q}} &\equiv \frac{4\pi I_1}{n_R c}S\,\frac{A\Xi_{\boldsymbol{0}}-\gamma_{\boldsymbol{q}}\Xi_{\boldsymbol{q}}}{\sqrt{1-\gamma_{\boldsymbol{q}}^2}},\\ F_0 &\equiv -\frac{4\pi I_1}{n_R c}AS^2\frac{N}{2}\Xi_{\boldsymbol{0}}, \end{aligned} \tag{12}$$

and we recall that, as discussed in Supplementary Note 1, $\gamma_{\boldsymbol{q}}\geq 0$. The quantities F_0, $f'_{\boldsymbol{q}}$, and $f_{\boldsymbol{q}}$ depend on the symmetries of the material, on the pump polarization, and they

are directly proportional to the integrated intensity of the pump field. The parameters $f'_{\boldsymbol{q}}$ measure the strengths of processes which conserve the number of magnons, while F_0 is a constant energy shift.

Crucially, the Hamiltonian $\hat{H}_1(t)$ includes processes which do not conserve the number of magnons. The strengths of these processes are measured by the parameters $f_{\boldsymbol{q}}$, and the existence of these terms is due to the fact that the Bogoliubov transformation of Eqs.(6) does not diagonalize simultaneously the equilibrium Hamiltonian and the Hamiltonian of light-matter interaction. To understand this point better, let us take a closer look at the expression of $f_{\boldsymbol{q}}$, for simplicity in the isotropic case ($A = 1$). In this case, we have

$$f_{\boldsymbol{q}} \propto \Xi_{\boldsymbol{q}} - \gamma_{\boldsymbol{q}} \Xi_{\boldsymbol{0}} = \sum_{\boldsymbol{\delta}} \mathrm{e}^{\mathrm{i}\boldsymbol{q}\cdot\boldsymbol{\delta}} \left[\Xi(\boldsymbol{\delta}) - \frac{1}{z} \sum_{\boldsymbol{\delta}'} \Xi(\boldsymbol{\delta}') \right] , \tag{13}$$

which clearly vanishes if $\Xi(\boldsymbol{\delta})$ is independent of $\boldsymbol{\delta}$, but is in general different from zero otherwise. Since $\Xi(\boldsymbol{\delta})$ depends on the direction of polarization of light, in the case of a linear polarization this quantity is indeed different in different directions (i.e., for different values of $\boldsymbol{\delta}$), which leads to non-vanishing $f_{\boldsymbol{q}}$ and two-magnon generation. This should be contrasted with the equilibrium exchange interaction, which in our cubic system is the same along all the crystal directions specified by the $\boldsymbol{\delta}$ vectors. The generation of two-magnon states in our model and experiment can thus be attributed to the fact that, while the equilibrium system has cubic symmetry, the non-equilibrium system has a lower symmetry because light introduces a preferential direction.

The intensity of the probe is much weaker than that of the pump. Therefore we will determine the modification of the dielectric tensor caused by the pump pulse only, as given by Eq.(1) of the main text. In the nearest-neighbour approximation for exchange, and by symmetry considerations [6], the dielectric tensor in our case reduces to

$$\epsilon_s^{\lambda\nu} \equiv \delta^{\lambda\nu} \sum_{\boldsymbol{R}} \sum_{\boldsymbol{\delta}} \sum_{\gamma} \rho_{\boldsymbol{\delta}}^{\lambda\gamma} \left\langle \hat{S}_{\boldsymbol{R}}^{\gamma\Uparrow} \hat{S}_{\boldsymbol{R}+\boldsymbol{\delta}}^{\gamma\Downarrow} \right\rangle, \tag{14}$$

with $\epsilon_s^{xx} = \epsilon_s^{yy}$, and in general $\epsilon_s^{xx} \neq \epsilon_s^{zz}$. To show that the time dependence of the rotation of the probe polarization is related to the magnon frequencies, we therefore need to compute the time-dependent spin correlation functions $\left\langle \hat{S}_{\boldsymbol{R}}^{\gamma\Uparrow} \hat{S}_{\boldsymbol{R}+\boldsymbol{\delta}}^{\gamma\Downarrow} \right\rangle$ after the pump excitation.

The state of the magnetic system after the action of the pump

The Schrödinger equation for the wave function $|\psi(t)\rangle$ of the magnetic system, neglecting the coupling with the probe, is

$$\mathrm{i}\frac{\mathrm{d}}{\mathrm{d}t}\left|\psi(t)\right\rangle = \left[\hat{H}_0 + \delta(t)\hat{F}\right]\left|\psi(t)\right\rangle . \tag{15}$$

Suppose that the system is in the ground state $|\psi_0\rangle$ for $t < 0$ (before the pump strikes), with $\hat{H}_0\left|\psi_0\right\rangle = 0$. Then the solution for $t > 0$ is [5]

$$\left|\psi(t)\right\rangle = \mathrm{e}^{-\mathrm{i}\hat{H}_0 t}\mathrm{e}^{-\mathrm{i}\hat{F}}\left|\psi_0\right\rangle . \tag{16}$$

Writing $\hat{F} = F_0 + \hat{F}_1$, it is clear that F_0 produces a constant phase factor which is irrelevant to compute observables for $t > 0$. We limit ourselves to considering the effect of the pump on the state of the system up to the lowest orders in its intensity, expanding Supplementary Eq.(16) in powers of $\hat{F}_1$ as

$$\begin{aligned}
\mathrm{e}^{\mathrm{i}F_0}\left|\psi(t)\right\rangle &\approx \mathrm{e}^{-\mathrm{i}\hat{H}_0 t}\left(1 - \mathrm{i}\hat{F}_1 - \frac{\hat{F}_1^2}{2}\right)\left|\psi_0\right\rangle \\
&= \left(1 - \frac{1}{2}\sum_{\boldsymbol{q}} f_{\boldsymbol{q}}^2\right)\left|\psi_0\right\rangle - \mathrm{i}\sum_{\boldsymbol{q}} f_{\boldsymbol{q}}\left[1 - \frac{\mathrm{i}}{2}\left(f'_{\boldsymbol{q}} + f'_{-\boldsymbol{q}}\right)\right]\mathrm{e}^{-\mathrm{i}2\omega_{\boldsymbol{q}}t}\hat{\alpha}^\dagger_{\boldsymbol{q}}\hat{\beta}^\dagger_{-\boldsymbol{q}}\left|\psi_0\right\rangle \\
&\quad - \frac{1}{2}\sum_{\boldsymbol{q}}\sum_{\boldsymbol{q}'} f_{\boldsymbol{q}} f_{\boldsymbol{q}'}\mathrm{e}^{-\mathrm{i}2\left(\omega_{\boldsymbol{q}}+\omega_{\boldsymbol{q}'}\right)t}\hat{\alpha}^\dagger_{\boldsymbol{q}}\hat{\beta}^\dagger_{-\boldsymbol{q}}\hat{\alpha}^\dagger_{\boldsymbol{q}'}\hat{\beta}^\dagger_{-\boldsymbol{q}'}\left|\psi_0\right\rangle .
\end{aligned} \tag{17}$$

The wave function in Supplementary Eq.(17) is normalized within an error of the order of $\left\langle\psi_0\right|\hat{F}_1^4\left|\psi_0\right\rangle$, which is of the fourth order in the pump intensity. In the following, we will neglect every quantity of order higher than 2 in the intensity of the pump. The state given in Supplementary Eq.(17) is a superposition of 0-, 2- and 4- magnon states (terms of higher orders in the intensity of the pump give contributions with higher numbers of magnons).

We now characterize the state of the system after the pump excitation by studying the time dependence of some relevant observables, neglecting the temperature. In the following, we will use the notation $X(t) \equiv \left\langle\psi(t)\right|\hat{X}\left|\psi(t)\right\rangle \equiv \left\langle\hat{X}(t)\right\rangle$.

Magnetic moments and antiferromagnetic vector

We derive the expressions for the local magnetic moments on the two magnetic sublattices, $\boldsymbol{S}_i^{\Uparrow}(t)$ and $\boldsymbol{S}_j^{\Downarrow}(t)$. The x and y components of the magnetic moments are zero (to all orders in the intensity of the pump), since each of them can be written as the expectation value over the zero-magnon state $|\psi_0\rangle$ of a sum of time-dependent operators involving odd numbers

of magnons. The z components, on the other hand, oscillate in time, with an oscillation amplitude which is at least of first order in the intensity of the pump, and $S_i^{z\Downarrow}(t) = -S_i^{z\Uparrow}(t)$. Therefore, despite the oscillation in time of the local magnetic moments, the total (macroscopic) magnetization $\boldsymbol{M}(t) = \boldsymbol{0}$, since for every i-th crystal cell $\boldsymbol{S}_i^{\Uparrow}(t) + \boldsymbol{S}_i^{\Downarrow}(t) = \boldsymbol{0}$. The total antiferromagnetic vector is

$$\boldsymbol{L}(t) \equiv \frac{N}{2}\left(\boldsymbol{S}_i^{\Uparrow}(t) - \boldsymbol{S}_i^{\Downarrow}(t)\right) = N\boldsymbol{S}_i^{\Uparrow}(t) \equiv \boldsymbol{u}^z L^z(t), \tag{18}$$

where $\boldsymbol{u}^z$ is the unit vector along the z direction, and

$$\begin{aligned} L^z(t) \approx & \left(NS + \frac{N}{2} - \sum_{\boldsymbol{k}} \frac{1}{\sqrt{1-\gamma_{\boldsymbol{k}}^2}}\right) - 2\sum_{\boldsymbol{k}} f_{\boldsymbol{k}}^2 \frac{1}{\sqrt{1-\gamma_{\boldsymbol{k}}^2}} \\ & - 2\sum_{\boldsymbol{k}} f_{\boldsymbol{k}} \frac{\gamma_{\boldsymbol{k}}}{\sqrt{1-\gamma_{\boldsymbol{k}}^2}} \left[\sin\left(2\omega_{\boldsymbol{k}} t\right) + \frac{1}{2}\left(f'_{\boldsymbol{k}} + f'_{-\boldsymbol{k}}\right)\cos\left(2\omega_{\boldsymbol{k}} t\right)\right], \end{aligned} \tag{19}$$

which is valid up to second order in the pump intensity. Since the magnon density of states dominates close to the highest frequency $\omega_{2M} \approx 2\,\omega_{\boldsymbol{k}}|_{\gamma_{\boldsymbol{k}}=0} = 2JSz$, we can expect that the main contributions to Supplementary Eq.(19) oscillate with the same frequency, and accordingly we can write the time-dependent part of $L^z(t)$ as

$$\Delta L^z(t) \approx A\sin\left(\omega_{2M} t\right) + B\cos\left(\omega_{2M} t\right), \tag{20}$$

where $A \approx -2\sum_{\boldsymbol{k}} f_{\boldsymbol{k}}\gamma_{\boldsymbol{k}}\left(1-\gamma_{\boldsymbol{k}}^2\right)^{-1/2}$ and $B \approx -\sum_{\boldsymbol{k}} f_{\boldsymbol{k}}\gamma_{\boldsymbol{k}}\left(1-\gamma_{\boldsymbol{k}}^2\right)^{-1/2}\left(f'_{\boldsymbol{k}} + f'_{-\boldsymbol{k}}\right)$. We can safely assume that $B << A$ and obtain Eq.(3) of the main text.

Energy and spin correlation functions

The pump provides to the magnetic system the energy needed to generate the two-magnon state. Afterwards, the energy of the magnetic system, to leading order in the pump intensity, is given by

$$E_{t>0} = \langle\psi(t)|\,\hat{H}_0\,|\psi(t)\rangle_{t>0} \approx \sum_{\boldsymbol{q}} 2\omega_{\boldsymbol{q}} f_{\boldsymbol{q}}^2, \tag{21}$$

and it *remains constant in time*, since we are neglecting the possible exchange of energy with reservoirs such as the phonon bath, or excitations of non-magnetic electronic degrees of freedom. While the total energy does not change in time, energy transfers occur between the magnetic degrees of freedom along different directions in the material.

We derive the *microscopic* spin correlation functions for pairs of sites (i, j) belonging to opposite aligned magnetic sublattices, neglecting all products of more than two

bosonic operators. Owing to spin isotropy in the plane orthogonal to the direction z of the magnetic moments, we have $\langle\psi(t)|\,\hat{S}_i^{y\Uparrow}\hat{S}_j^{y\Downarrow}\,|\psi(t)\rangle = \langle\psi(t)|\,\hat{S}_i^{x\Uparrow}\hat{S}_j^{x\Downarrow}\,|\psi(t)\rangle$, while in general $\langle\psi(t)|\,\hat{S}_i^{x\Uparrow}\hat{S}_j^{x\Downarrow}\,|\psi(t)\rangle \neq \langle\psi(t)|\,\hat{S}_i^{z\Uparrow}\hat{S}_j^{z\Downarrow}\,|\psi(t)\rangle$. We define the *macroscopic* spin correlation functions as the sums of these microscopic quantities over all the magnetic sites of the system, which result in

$$\begin{aligned}
&\sum_{\langle i,j\rangle}\langle\psi(t)|\,\hat{S}_i^{z\Uparrow}\hat{S}_j^{z\Downarrow}\,|\psi(t)\rangle \\
&\approx -S(S+1)\frac{N}{2}z + Sz\sum_{\boldsymbol{q}}\frac{1}{\sqrt{1-\gamma_{\boldsymbol{q}}^2}} + 2Sz\sum_{\boldsymbol{q}} f_{\boldsymbol{q}}^2\frac{1}{\sqrt{1-\gamma_{\boldsymbol{q}}^2}} \\
&\quad + 2Sz\sum_{\boldsymbol{q}} f_{\boldsymbol{q}}\frac{\gamma_{\boldsymbol{q}}}{\sqrt{1-\gamma_{\boldsymbol{q}}^2}}\left[\sin\left(2\omega_{\boldsymbol{q}}t\right) + \frac{1}{2}\left(f'_{\boldsymbol{q}} + f'_{-\boldsymbol{q}}\right)\cos(2\omega_{\boldsymbol{q}}t)\right],
\end{aligned} \tag{22}$$

$$\begin{aligned}
&\sum_{\langle i,j\rangle}\langle\psi(t)|\,\hat{S}_i^{x\Uparrow}\hat{S}_j^{x\Downarrow}\,|\psi(t)\rangle = \sum_{\langle i,j\rangle}\langle\psi(t)|\,\hat{S}_i^{y\Uparrow}\hat{S}_j^{y\Downarrow}\,|\psi(t)\rangle \\
&\approx -\frac{1}{2}Sz\sum_{\boldsymbol{q}}\frac{\gamma_{\boldsymbol{q}}^2}{\sqrt{1-\gamma_{\boldsymbol{q}}^2}} - Sz\sum_{\boldsymbol{q}} f_{\boldsymbol{q}}^2\frac{\gamma_{\boldsymbol{q}}^2}{\sqrt{1-\gamma_{\boldsymbol{q}}^2}} \\
&\quad - Sz\sum_{\boldsymbol{q}} f_{\boldsymbol{q}}\frac{\gamma_{\boldsymbol{q}}}{\sqrt{1-\gamma_{\boldsymbol{q}}^2}}\left[\sin\left(2\omega_{\boldsymbol{q}}t\right) + \frac{1}{2}\left(f'_{\boldsymbol{q}} + f'_{-\boldsymbol{q}}\right)\cos(2\omega_{\boldsymbol{q}}t)\right],
\end{aligned} \tag{23}$$

where we keep only contributions up to the second order in the intensity of the pump. At the first order in the intensity, the pump alters the correlation functions via a time-dependent contribution (included in the $f_{\boldsymbol{q}}$ parameters) which provides components that oscillate with the frequencies of the excited two-magnon modes (sin-like). One can immediately check that the total correlation function $\sum_{\langle i,j\rangle}\langle\psi(t)|\,\hat{\boldsymbol{S}}_i^{\Uparrow}\cdot\hat{\boldsymbol{S}}_j^{\Downarrow}\,|\psi(t)\rangle$ is time-independent, since all the time-dependent terms cancel out in the sum over (x, y, z), while the term quadratic in the intensity is $\sum_{\boldsymbol{q}} f_{\boldsymbol{q}}^2\,(2\omega_{\boldsymbol{q}}/J)$. Multiplying by J yields the two-magnon term in the energy of the magnetic system for $t > 0$, Supplementary Eq.(21).

The time-dependent modulation of the spin correlation functions along three orthogonal directions lasts after the pump is gone, despite the fact that the energy of the system is constant. This in turn modulates the dielectric tensor in time, according to Eq.(1) of the main text.

Macroscopic antiferromagnetic vector and spin correlation function

While the mechanism of light-matter interaction in our experiment is intrinsically microscopic, since it affects the short-ranged exchange interactions of the material, the coherence of the pump pulse determines a modulation in time of the *macroscopic* antiferromagnetic vector $\boldsymbol{L}(t)$. While the mechanism of exchange modulation alone (i.e., in the absence of spin-orbit coupling) cannot produce a precession of $\boldsymbol{L}(t)$, the size of the vector changes in time, according to Supplementary Eq.(19).

By comparing Eqs.(19) and (22), it can be seen that the time-dependent parts of $L^z(t)$ and of the correlation function $\sum_{\langle i,j\rangle} \langle\psi(t)|\, \hat{S}_i^{z\Uparrow}\hat{S}_j^{z\Downarrow}\,|\psi(t)\rangle$ are proportional. This can be explained as follows. Denoting as $\boldsymbol{R}$ the sites of the $\Uparrow$ sublattice, we write

$$\begin{aligned}\sum_{\langle i,j\rangle} \langle\psi(t)|\, \hat{S}_i^{z\Uparrow}\hat{S}_j^{z\Downarrow}\,|\psi(t)\rangle =&\sum_{\boldsymbol{R}}\sum_{\boldsymbol{\delta}} \langle\psi(t)|\, \hat{S}_{\boldsymbol{R}}^{z\Uparrow}\hat{S}_{\boldsymbol{R}+\boldsymbol{\delta}}^{z\Downarrow}\,|\psi(t)\rangle \\ =&-zS^2\frac{N}{2}+S\sum_{\boldsymbol{R}}\sum_{\boldsymbol{\delta}} \langle\psi(t)|\Big(\hat{b}_{\boldsymbol{R}}^{\dagger}\hat{b}_{\boldsymbol{R}}+\hat{a}_{\boldsymbol{R}+\boldsymbol{\delta}}^{\dagger}\hat{a}_{\boldsymbol{R}+\boldsymbol{\delta}}\Big)|\psi(t)\rangle \\ &-\sum_{\boldsymbol{R}}\sum_{\boldsymbol{\delta}} \langle\psi(t)|\, \hat{b}_{\boldsymbol{R}}^{\dagger}\hat{b}_{\boldsymbol{R}}\hat{a}_{\boldsymbol{R}+\boldsymbol{\delta}}^{\dagger}\hat{a}_{\boldsymbol{R}+\boldsymbol{\delta}}\,|\psi(t)\rangle\,. \end{aligned} \tag{24}$$

The term in the last line of Supplementary Eq.(24) should be neglected in the framework of our non-interacting theory. The antiferromagnetic vector can similarly be written as

$$\begin{aligned}L^z(t) =&\sum_{\boldsymbol{R}} \langle\psi(t)|\,\Big(\hat{S}_{\boldsymbol{R}}^{z\Uparrow}-\hat{S}_{\boldsymbol{R}+\boldsymbol{\delta}_1}^{z\Downarrow}\Big)\,|\psi(t)\rangle \\ =&SN-\sum_{\boldsymbol{R}} \langle\psi(t)|\Big(\hat{b}_{\boldsymbol{R}}^{\dagger}\hat{b}_{\boldsymbol{R}}+\hat{a}_{\boldsymbol{R}+\boldsymbol{\delta}_1}^{\dagger}\hat{a}_{\boldsymbol{R}+\boldsymbol{\delta}_1}\Big)|\psi(t)\rangle \\ =&SN-\frac{1}{z}\sum_{\boldsymbol{R}}\sum_{\boldsymbol{\delta}} \langle\psi(t)|\Big(\hat{b}_{\boldsymbol{R}}^{\dagger}\hat{b}_{\boldsymbol{R}}+\hat{a}_{\boldsymbol{R}+\boldsymbol{\delta}}^{\dagger}\hat{a}_{\boldsymbol{R}+\boldsymbol{\delta}}\Big)|\psi(t)\rangle\,, \end{aligned} \tag{25}$$

where $\boldsymbol{\delta}_1$ is one of the six nearest-neighbour vectors $\{\boldsymbol{\delta}\}$, namely the one that we choose to define the magnetic unit cell (so that the cell containing $\boldsymbol{R}$ consists of the magnetic sites at positions $\boldsymbol{R}$ and $\boldsymbol{R}+\boldsymbol{\delta}_1$). We then see that

$$\sum_{\langle i,j\rangle} \langle\psi(t)|\, \hat{S}_i^{z\Uparrow}\hat{S}_j^{z\Downarrow}\,|\psi(t)\rangle \approx zS^2\frac{N}{2}-zSL^z(t). \tag{26}$$

We have then demonstrated that, in the framework of non-interacting magnons, the time-dependent part of the spin correlation function along z (which is in turn related to the spin correlation functions along x and y, since their sum is time-independent after the action of

the pump) is equal to the time-dependent part of the antiferromagnetic vector $L^z(t)$, apart from a negative proportionality constant. This shows that controlling the short-ranged spin correlation functions of the material in a coherent way by means of light allows to control the size of the macroscopic antiferromagnetic vector.

Supplementary Note 3

In Fig. 2(**b**) of the main text we report the probe polarization dynamics as a function of the pump-probe delay, performed for two orthogonal polarizations of the probe. By rotating the polarization of the probe beam the sign of the 2M oscillation is reversed (i.e. π phase shift). If the signal had originated from the Faraday effect, the two time traces shown in Fig. 2(**b**) of the main text would have exhibited the same phase. In fact, the Faraday effect is defined by the antisymmetric components of the dielectric tensor [7]. We therefore conclude that the measured signal can only be related to the symmetric components of the dielectric tensor [6, 7]. As mentioned in the main text, the dynamics of the two-magnon mode can be detected only by a magneto-optical effect quadratic in the spin deviation, like the antiferromagnetic linear dichroism. The dynamics of this effect is proportional to the time-evolution of the spin correlation function. The microscopic theory derived in Supplementary Note 2 predicts that the time-dependent parts of the z- and x- components of the spin correlation function differ in sign and amplitude [see Supplementary Eqs.(22) and (23)]. This is in excellent agreement with the data shown in Fig. 2(**b**) of the main text, where the oscillations detected with perpendicular probe polarizations show opposite sign and different amplitude.

Supplementary Note 4

The cubic Heisenberg antiferromagnet $KNiF_3$ belongs to the $m3m$ point group [8], therefore the light-matter interaction describing the two-magnon process is expressed by the following thermodynamical potential [1]:

$$\Phi = \chi^{ijkl}_{\alpha\alpha\beta\beta} E^\alpha_i E^\alpha_j \left\langle \hat{S}^\beta_k \hat{S}^\beta_l \right\rangle \equiv \chi_{\alpha\alpha\beta\beta} E^\alpha E^\alpha \left\langle \hat{S}^{\beta\Uparrow} \hat{S}^{\beta\Downarrow} \right\rangle, \tag{27}$$

where $\alpha, \beta \in \{x, y, z\}$ are cartesian coordinates and the indices i, j, k, l are position labels. In Supplementary Eq.(27), $\chi^{ijkl}_{\alpha\alpha\beta\beta}$ is a fourth-rank polar tensor, $E^{\alpha}_{i,j}$ are the components of the electric field of the pump beam at the positions i or j, and $\left\langle \hat{S}^{\beta}_{k} \hat{S}^{\beta}_{l} \right\rangle$ is the correlation function between components of the spins at sites k and l. For simplicity of notation, in this section we will drop the position indices as in the last passage of Supplementary Eq.(27). The magnetic order of $KNiF_3$ is properly described by a nearest-neighbour Heisenberg exchange interaction [8]. Considering the geometry reported in Fig. 1(**b**) of the main text, from Supplementary Eq.(27) we can calculate the interaction energy corresponding to different polarizations of the pump. If the electric field of the excitation beam is oriented along the x-axis, we get

$$\Phi^{(\|x)} = \chi_{xxxx} E^x E^x \left\langle \hat{S}^{x\Uparrow} \hat{S}^{x\Downarrow} \right\rangle + \chi_{xxyy} E^x E^x \left\langle \hat{S}^{y\Uparrow} \hat{S}^{y\Downarrow} \right\rangle + \chi_{xxzz} E^x E^x \left\langle \hat{S}^{z\Uparrow} \hat{S}^{z\Downarrow} \right\rangle, \qquad (28)$$

while if the pump is polarized along the z-axis we obtain

$$\Phi^{(\|z)} = \chi_{zzzz} E^z E^z \left\langle \hat{S}^{z\Uparrow} \hat{S}^{z\Downarrow} \right\rangle + \chi_{zzyy} E^z E^z \left\langle \hat{S}^{y\Uparrow} \hat{S}^{y\Downarrow} \right\rangle + \chi_{zzxx} E^z E^z \left\langle \hat{S}^{x\Uparrow} \hat{S}^{x\Downarrow} \right\rangle. \qquad (29)$$

The components of χ satisfy the symmetries of the $m3m$ point group [9], namely $\chi_{xxxx} = \chi_{yyyy} = \chi_{zzzz}$ and $\chi_{xxyy} = \chi_{yyxx} = \chi_{yyzz} = \chi_{zzyy} = \chi_{zzxx} = \chi_{xxzz}$. Moreover, the following relations hold for the spin correlation functions (see the derivation in section 3): $\left\langle \hat{S}^{x\Uparrow} \hat{S}^{x\Downarrow} \right\rangle = \left\langle \hat{S}^{y\Uparrow} \hat{S}^{y\Downarrow} \right\rangle \neq \left\langle \hat{S}^{z\Uparrow} \hat{S}^{z\Downarrow} \right\rangle$, where z is the direction parallel to the spin alignment. We can then re-write Supplementary Eqs.(28) and (29) as

$$\Phi^{(\|x)} = \left(\chi_{xxxx} + \chi_{xxyy}\right) E^x E^x \left\langle \hat{S}^{x\Uparrow} \hat{S}^{x\Downarrow} \right\rangle + \chi_{xxyy} E^x E^x \left\langle \hat{S}^{z\Uparrow} \hat{S}^{z\Downarrow} \right\rangle, \qquad (30)$$

$$\Phi^{(\|z)} = \chi_{xxxx} E^z E^z \left\langle \hat{S}^{z\Uparrow} \hat{S}^{z\Downarrow} \right\rangle + 2\chi_{xxyy} E^z E^z \left\langle \hat{S}^{x\Uparrow} \hat{S}^{x\Downarrow} \right\rangle. \qquad (31)$$

The two expressions derived above show that the excitations of the spin system provided by x- and z-polarized pump beams are not equivalent. This is in agreement with the data shown in Fig. 4(**a**) of the main text, in which these two polarization states trigger oscillations with a phase difference equal to π.

Supplementary Note 5

One of the possible approaches to the time-frequency analysis consists in the evaluation of the Wigner transform of the signal at each delay time. In this framework, we rely on the

following definition[10],

$$W(t,\nu,\delta t) \equiv \left| \int U(t') \exp\left[-2\pi \left(\frac{t-t'}{\delta t} \right)^2 \right] \exp\left(-i2\pi\nu t'\right) dt' \right|^2, \tag{32}$$

where $W(t,\nu,\delta t)$ reveals the frequencies and amplitudes of the oscillatory components of the time-dependent signal U, at a certain delay time t. According to this definition, the data have to be multiplied by a Gaussian function, with an arbitrary width (δt), centred at a delay time t. The result of this multiplication has then to be Fourier transformed, providing the spectrum of the signal U at the delay time t. Note that in this well-established procedure [10, 11] the only parameter which is set by the user is the width of the Gaussian function, which has to be chosen considering the frequency and the lifetime of the oscillating signal. We chose $\delta t = 100$ fs.

In Fig. 5(**b**) of the main text the power Wigner transform of the data shown in Fig. 2(**a**) is reported. The 2M frequency is modulated at 7.5 THz [see inset of Fig. 5(**b**) of the main text]. The nature of this modulation was unraveled via the analysis of the temperature dependence of the effect. We applied the Wigner transform to the time traces measured for different temperatures (see Fig. 3 in the main text). The modulation frequency is plotted as a function of the temperature in Supplementary Fig. 2, which shows that the frequency is independent of temperature, substantiating the identification of the modulation with the lattice stretching mode of $KNiF_3$. Note that although we measured the ultrafast dynamics of the spin system up to the Néel point ($T_N = 246$ K [12]), only data up to 190 K were considered in Supplementary Fig. 2. The reason is that the lifetime of the magnetic oscillations, for temperatures equal to or higher than 200 K, is too short to reveal a 7.5 THz (period $\approx$ 130 fs) modulation.

Supplementary References

[1] M.G. Cottam and D.J. Lockwood, *Light Scattering in Magnetic Solids*, 1st ed. (Wiley-Interscience, 1986).

[2] F. Ganot, C. Dugautier, P. Moch and J. Nouet, *Brillouin scattering and ultrasonic propagation in the nearly cubic antiferromagnet $KNiF_3$*, Journal of Physics C: Solid State **15,** 801-823 (1982).

[3] A. Auerbach, *Interacting Electrons and Quantum Magnetism* 1st ed. (Springer, 1998).

[4] U. Balucani and V. Tognetti, *Theory of Two-Magnon Raman Scattering in the Ordered Region for Cubic Antiferromagnets*, Physical Review B **8,** 4247-4257 (1973).

[5] J. Zhao, A. V. Bragas, D.J. Lockwood and R. Merlin, *Magnon squeezing in antiferromagnetic MnF_2 and FeF_2*, Physical Review B **73**, 184434 (2006).

[6] J. Ferré and G. A. Gehring, *Linear optical birefringence of magnetic crystals*, Reports on Progress in Physics **47,** 513-611 (1984).

[7] A.K. Zvezdin and V.A. Kotov, *Modern Magneto-optics and Magneto-optical Materials* 1st ed. (Taylor and Francis, 1997).

[8] M. Lines, *Comparative Studies of Magnetism in $KNiF_3$ and K_2NiF_4*, Phys. Rev. **164,** 736-748 (1967).

[9] R. Birss, *Symmetry and Magnetism* 2nd ed.,(Wiley Sons, New York, 1966).

[10] H. Bartel, K.-H. Brenner and A. Lohmann, *The Wigner distribution function and its optical production*, Optics Communications **32,** 32-38 (1980).

[11] A. Gambetta et al. *Real-time observation of nonlinear coherent phonon dynamics in single-walled carbon nanotubes*, Nature Physics **2,** 515-520 (2006).

[12] D. Bossini, A.M. Kalashnikova, R.V. Pisarev, T. Rasing and A.V. Kimel, *Controlling coherent and incoherent spin dynamics by steering the photoinduced energy flow*, Physical Review B **89,** 060405 (R) (2014).
